# Dynamic Lactate-to-Albumin Ratio Trajectories and Outcomes in Sepsis-Associated Acute Kidney Injury: Evidence From MIMIC-IV and a Multicenter Chinese Cohort

**DOI:** 10.2196/92838

**Published:** 2026-08-14

**Authors:** Fei Li, Xiaoxia Guo, Yunhao Li, Jin Zhang, Jingyi Wang, Pengchao Tian, Xi Zheng, Lifeng Huang, Yue Zheng, Wenxiong Li, Na Cui

**Affiliations:** 1 Department of Critical Care Medicine, Beijing Chao-Yang Hospital, Capital Medical University, No. 8, Gongti South Road, Chaoyang District, Beijing, 100020, China, 86 85231458

**Keywords:** sepsis-associated acute kidney injury, lactate-to-albumin ratio, trajectory modeling, mortality, external validation

## Abstract

**Background:**

Sepsis-associated acute kidney injury (SA-AKI) is a frequent and life-threatening complication of sepsis. While the static lactate-to-albumin ratio (LAR) has prognostic value, its dynamic temporal evolution during early resuscitation and its utility for guiding clinical risk stratification remain underexplored.

**Objective:**

The aim of the study is to identify distinct dynamic trajectories of LAR, evaluate their independent associations with adverse clinical outcomes, and construct a practical prognostic nomogram for patients with SA-AKI.

**Methods:**

Data were extracted from the Medical Information Mart for Intensive Care IV database, including adult patients with SA-AKI and ≥ 3 lactate and albumin measurements within the first 72 hours of intensive care unit admission. A multicenter external validation cohort was assembled from 5 tertiary hospitals in Beijing, China. Group-based trajectory modeling identified distinct LAR trajectories. The primary outcome was 28-day mortality; secondary outcomes included 90-day mortality and continuous renal replacement therapy initiation. Trajectory-outcome associations were assessed using multivariable Cox and logistic regression models. Robustness was examined using restricted cubic splines, inverse probability of treatment weighting, weight truncation, doubly robust estimation, and subgroup analyses. Incremental predictive value beyond single baseline LAR was quantified, and a trajectory-integrated nomogram was developed and externally validated.

**Results:**

Among the 615 patients in the primary cohort, 3 LAR trajectories were identified: trajectory 1 (low-stable type), trajectory 2 (rapid-clearance type), and trajectory 3 (delayed-clearance type). In adjusted multivariable models, trajectory 3 (vs trajectory 1) was independently associated with increased 28-day mortality risk (hazard ratio 1.63, 95% CI 1.00‐2.64; *P*=.0496), 90-day mortality (hazard ratio 1.72, 95% CI 1.14‐2.60; *P*=.01), and continuous renal replacement therapy initiation (odds ratio 3.40, 95% CI 1.64‐7.15; *P*=.001). Conversely, the mortality risk for trajectory 2 did not differ significantly from trajectory 1. Sensitivity analyses supported these findings, although trajectory 3 associations attenuated in inverse probability of treatment weighting–weighted analyses. Incorporating trajectories improved predictive accuracy over single baseline LAR (continuous net reclassification improvement 0.170, integrated discrimination improvement 0.023; both *P*=.01). In the external validation cohort (n=508), 3 analogous trajectories were identified. Trajectory 3 consistently conferred a higher mortality risk across both cohorts, whereas trajectory 2 was significantly associated with 28-day mortality only in validation. The nomogram exhibited modest discrimination in the external cohort (concordance index=0.612), acceptable calibration, and potential utility as a supplementary bedside risk stratification tool.

**Conclusions:**

Dynamic LAR trajectories are independently associated with 28- and 90-day mortality in SA-AKI. The delayed-clearance trajectory identifies a specific high-risk phenotype, suggesting that longitudinal LAR monitoring and the constructed nomogram may support early risk stratification and inform clinical decision-making.

## Introduction

Sepsis-associated acute kidney injury (SA-AKI) is a frequent and devastating complication in critically ill patients, associated with increased risks of chronic kidney disease, cardiovascular events, and mortality [1]. The pathophysiology of SA-AKI is multifactorial, involving microcirculatory dysfunction, metabolic reprogramming, immune and inflammatory dysregulation, and tubular epithelial injury and impaired repair [2,3]. Current management focuses on early recognition, antimicrobial therapy, appropriate fluid resuscitation, vasopressor support, and initiation of renal replacement therapy when indicated for severe cases [4,5]. Traditional functional markers, such as serum creatinine and urine output, demonstrate low sensitivity and significant lag for early detection of SA-AKI. Therefore, identifying novel biomarkers for early diagnosis and prognostic assessment in SA-AKI remains crucial. Consistent with this concept, the Acute Disease Quality Initiative 23 Consensus Conference emphasized that acute kidney injury (AKI) risk assessment should move beyond traditional functional indices alone and incorporate additional biologically informative markers to improve early risk prediction [6].

The prognostic impact of inflammatory mediators, endothelial injury markers, and metabolic indicators in SA-AKI has been widely investigated. Lactate, a byproduct of anaerobic metabolism, serves as a biomarker reflecting tissue hypoperfusion and impaired oxygen use [7]. Elevated lactate levels have been associated with poor outcomes and markedly increased mortality in patients with septic shock [8]. However, as a single metabolic marker, lactate is influenced by multiple factors, including medications (eg, metformin and long-acting β-blockers), vasoactive therapy, and hepatic or renal dysfunction [9-12]. Albumin, the most abundant plasma protein, maintains colloid osmotic pressure, facilitates hormone and drug transport, acts as a pH buffer, and serves as a negative acute-phase reactant, decreasing during inflammation or stress [13]. Recent studies have demonstrated that hypoalbuminemia significantly increases both short-term and long-term mortality in patients with sepsis, serving as a prognostic biomarker [14,15]. In addition, hypoalbuminemia has been shown to be associated with worse outcomes in patients with SA-AKI [16]. However, similar to lactate levels, albumin levels are susceptible to multiple factors, including chronic underlying diseases, nutritional status, hepatic function, and changes in capillary permeability [17]. Thus, using lactate or albumin alone as a prognostic marker has limitations.

The lactate-to-albumin ratio (LAR) integrates information on tissue hypoperfusion (lactate) and nutritional or inflammatory status (albumin) and has attracted increasing attention as a prognostic marker in sepsis. Several studies have shown that LAR performs better than either lactate or albumin alone in predicting short-term and long-term mortality, as well as severe outcomes such as the need for renal replacement therapy or mechanical ventilation [18,19]. Moreover, LAR has been identified as an early biomarker for SA-AKI, outperforming traditional severity scores and laboratory parameters in predicting adverse outcomes and serving as an independent risk factor for short-term mortality [20,21]. These findings suggest that LAR may serve as a simple and emerging prognostic biomarker for SA-AKI with practical value in early risk prediction. However, it must be recognized that the development and progression of SA-AKI represent a continuously evolving pathophysiological process. Most existing studies are limited to evaluating LAR at a single time point, such as at admission or diagnosis, making it difficult to capture its dynamic evolution during the early phase of critical illness and resuscitation. Therefore, dynamically monitoring the trajectory of LAR may more accurately reflect the progression of SA-AKI, potentially offering incremental prognostic value over static baseline measurements. Nevertheless, clinical studies in this area remain scarce, and practical tools integrating these dynamic patterns for bedside decision-making are currently lacking.

Group-based trajectory modeling (GBTM), a type of finite mixture modeling or latent class trajectory modeling, is a statistical approach used to identify latent phenotypes with similar longitudinal patterns [22]. GBTM enables the identification of distinct trajectories of clinical or biological markers over time, often improving outcome prediction compared with single-point measurements [23]. The Medical Information Mart for Intensive Care IV (MIMIC-IV) database provides comprehensive clinical data on critically ill patients, offering a valuable resource for such analyses [24]. Based on the MIMIC-IV (version 2.2) database and supplemented by an external multicenter validation cohort, this study applied GBTM to characterize dynamic LAR trajectories among patients with SA-AKI and to examine their associations with clinical outcomes. Furthermore, we aimed to systematically quantify the incremental predictive value of these dynamic trajectories compared with a single baseline LAR and to construct and externally validate a clinical prognostic nomogram, ultimately providing a practical tool to improve early risk stratification in critically ill populations. The reporting of this cohort study complies with the STROCSS (Strengthening the Reporting of Cohort, Cross-Sectional and Case-Control Studies in Surgery) 2025 guidelines [25].

## Methods

### Data Source and Study Design

Data for this study were obtained from the MIMIC-IV (version 2.2) database [24], which contains deidentified information from over 50,000 intensive care unit (ICU) admissions at the Beth Israel Deaconess Medical Center between 2008 and 2019. The database includes detailed records of demographics, laboratory tests, medications, surgical procedures, and survival outcomes. This study involved an external validation cohort derived from a multicenter, prospective observational cohort study conducted between June 2023 and October 2025 at 5 tertiary hospitals in Beijing, China (Peking Union Medical College Hospital, Beijing Shijitan Hospital, Beijing Jishuitan Hospital, Beijing Hospital, and Beijing Chaoyang Hospital). The inclusion and exclusion criteria for the external validation cohort were identical to those applied to the MIMIC-IV cohort.

This cohort study included only patients with SA-AKI from the MIMIC-IV (version 2.2) database who met the following criteria: first hospital admission, first ICU admission, ICU length of stay ≥72 hours, age ≥18 years, a confirmed diagnosis of SA-AKI on the day of ICU admission, and availability of lactate and albumin measurements at least 3 times within the predefined 0-, 24-, 48-, and 72-hour time windows following ICU admission. SA-AKI was defined according to established criteria [1], requiring both Sepsis-3.0 criteria (presence of infection with Sequential Organ Failure Assessment [SOFA] score ≥2) and AKI diagnostic criteria (meeting Kidney Disease Improving Global Outcomes [KDIGO] criteria). To clarify the temporal sequence, the Sepsis-3.0 criteria had to be met concurrently with or prior to the KDIGO criteria, with AKI occurring within 7 days of sepsis onset. Exclusion criteria included: (1) end-stage renal disease or dialysis dependence, (2) pregnancy or lactation, (3) AIDS, (4) patients with burns, (5) end-stage malignancy, and (6) patients with organ transplants.

### Ethical Considerations

This study used data from 2 separate cohorts, adhering strictly to privacy and confidentiality protocols, with no financial compensation provided to participants and no identifiable information or images included in this paper. For the primary analysis, the use of the preanonymized and deidentified MIMIC-IV database was approved by the institutional review boards of the Massachusetts Institute of Technology and Beth Israel Deaconess Medical Center. Individual informed consent was waived for this secondary analysis under the HIPAA (Health Insurance Portability and Accountability Act) Safe Harbor provision, and investigators accessed the data after obtaining Collaborative Institutional Training Initiative certification (certificate 57230846). The external prospective validation cohort, registered with the Chinese Clinical Trial Registry (ChiCTR2300074175), was reviewed and approved by the ethics committees of Peking Union Medical College Hospital (approval K3148, I-22PJ1104), Beijing Shijitan Hospital (IIT2023-007-002), Beijing Jishuitan Hospital (K2023-195-00), Beijing Hospital (2023BJYYEC-150‐01), and Beijing Chaoyang Hospital (2025-Ke-869). For this validation cohort, written informed consent was obtained from all patients or their legally authorized representatives prior to data collection, and all extracted data were strictly anonymized and stored on secure, encrypted servers.

### Data Extraction

Based on the inclusion and exclusion criteria, data were extracted using Navicat Premium 15 through Structured Query Language queries, yielding a total of 615 patients with SA-AKI in the MIMIC-IV (version 2.2) database. The external validation cohort ultimately included 508 eligible patients with SA-AKI. Baseline variables, including severity scoring systems (Acute Physiology and Chronic Health Evaluation III [APACHE III], Simplified Acute Physiology Score II [SAPS II], and SOFA) and therapeutic interventions (broad-spectrum antibiotic use and binary vasopressor use), were rigorously defined as data documented exclusively within the first 24 hours of ICU admission. For continuous renal replacement therapy (CRRT), only a binary indicator of whether CRRT was initiated at any time during the ICU stay was available, without the exact timestamp of initiation. Therefore, CRRT was treated as a postadmission clinical event variable rather than a baseline covariate. The analytical handling and associated temporal limitations of the CRRT outcome are detailed in the Statistical Analysis and Strengths and Limitations sections, respectively.

To minimize bias caused by inconsistent sampling times, lactate and albumin values were standardized using a time-window approach in which 4 reference points were defined at 0, 24, 48, and 72 hours after ICU admission, and each point allowed a ±12-hour collection window. To address potential overlapping boundaries at 12, 36, and 60 hours, an explicit data-handling rule was applied: if multiple laboratory values were drawn within a window or fell exactly on an overlapping boundary, the measurement recorded closest to the exact reference hour (0, 24, 48, or 72 hours) was selected. Only patients with both lactate and albumin values available in at least 3 distinct windows were included in the final cohort. Prior to trajectory modeling, continuous variables, including LAR, were winsorized at the 1st and 99th percentiles to mitigate the influence of extreme outliers.

Data collected in this study included demographic information (age, sex, race, and BMI), vital signs (temperature, heart rate, respiratory rate, oxygen saturation, systolic blood pressure, diastolic blood pressure, and mean arterial pressure), laboratory parameters (white blood cell [WBC] count, platelet count, hemoglobin, hematocrit, lymphocytes, monocytes, neutrophils, glucose, serum creatinine, blood urea nitrogen, anion gap [AG], bicarbonate [HCO_3_^−^], sodium [Na^+^], potassium [K^+^], calcium [Ca²^+^], chloride [Cl⁻], international normalized ratio [INR], prothrombin time [PT], activated partial thromboplastin time, alanine aminotransferase [ALT], alkaline phosphatase, aspartate aminotransferase [AST], total bilirubin [TBIL], and lactate dehydrogenase), ICU scoring systems (APACHE III, SAPS II, SOFA, and Charlson Comorbidity Index), comorbidities (hypertension, diabetes mellitus, coronary artery disease, heart failure [HF], chronic obstructive pulmonary disease, and liver disease [including cirrhosis and liver failure]), therapeutic interventions (vasopressor use, mechanical ventilation, CRRT, broad-spectrum antibiotic use, exogenous albumin administration, and fluid resuscitation [≥30 mL/kg]), and outcome indicators (hospital and ICU length of stay, in-hospital, 28-day, and 90-day mortality).

The primary exposure variable was the dynamic trajectory of the LAR during ICU stay, derived from serial measurements of lactate and albumin.

### Follow-Up and Outcomes

The primary outcome was 28-day all-cause mortality. The secondary outcomes included 90-day all-cause mortality and CRRT initiation. For both the Medical Information Mart for Intensive Care (MIMIC) and external hospital cohorts, follow-up began at ICU admission in this study. All 28- and 90-day mortality outcomes were calculated from this index time. Patients who were discharged alive and had no subsequent death records were considered alive for the corresponding follow-up window, and no additional adjustments were made for the date of hospital discharge.

### Statistical Analysis

LAR was calculated based on lactate and albumin measurements at the 4 time windows. In the MIMIC-IV cohort, the proportions of missing LAR values at 0, 24, 48, and 72 hours were 5.85% (36/615), 10.73% (66/615), 11.71% (72/615), and 14.96% (92/615), respectively. Corresponding proportions in the external validation cohort were 11.42% (58/508), 7.48% (38/508), 5.12% (26/508), and 13.19% (67/508). To handle missing data at later time points (partly due to early death or ICU discharge), GBTM was performed using the R package *lcmm* [26]. Within this framework, missing repeated measures were statistically managed using full information maximum likelihood. This method allows all available data points to inform the model without removing patients with incomplete trajectories, thereby reducing potential bias from incomplete repeated measurements. Trajectory modeling used cubic polynomial functions for LAR trajectories, and models with 1 to 7 classes were fitted to select the optimal number of classes. The optimal model was determined based on comprehensive evaluation using the following criteria [27]: (1) Bayesian information criterion and Akaike information criterion, with lower values indicating better fit; (2) average posterior probability (AvgPostProb) >0.7 to ensure classification reliability; (3) minimum sample proportion for each trajectory group not less than 5%‐10%; (4) entropy >0.7 indicating good between-group discrimination; and (5) clinical interpretability of the model.

Baseline covariates with missing rates ≥20% were excluded, whereas missing values in the remaining baseline covariates were imputed using multiple imputation with the random forest algorithm implemented via the *mice* package in R. The normality of continuous variables was assessed using the Shapiro-Wilk test. Normally distributed variables were expressed as mean (SD) and compared using one-way ANOVA, whereas nonnormally distributed variables were expressed as median (IQR) and compared using the Kruskal-Wallis test. Categorical variables were presented as counts (%) and compared using the chi-square test or Fisher exact test, as appropriate.

Survival analyses were performed using Kaplan-Meier curves and multivariable Cox proportional hazards models to examine associations between LAR trajectory classes and mortality. To address confounding by artificial clearance mechanisms while maintaining the clinical simplicity of the prognostic tools, exogenous albumin administration was strictly adjusted as a baseline fixed covariate in the mortality models. CRRT was not included as a covariate in the mortality models and was analyzed separately as a secondary outcome. CRRT initiation was treated as the dependent variable in this analysis. Due to the lack of precise initiation timing, this analysis was not intended to infer time-sequenced causal effects and should be interpreted as an association. Covariates for multivariable models were predefined based on clinical rationale and core interventions highlighted during peer review. Three stepwise models were constructed: model A (unadjusted), model B (partially adjusted for key clinical interventions altering LAR and hemodynamics: baseline liver disease, exogenous albumin administration, and fluid resuscitation target ≥30 mL/kg), and Model C (fully adjusted). For 28- and 90-day mortality outcomes, model C further adjusted for APACHE III score, AKI stage, HF, and vasopressor use. For CRRT initiation, multivariable logistic regression was used, with model C additionally adjusting for model B covariates plus AKI stage, SOFA score, vasopressor use, and AG. Furthermore, the variance inflation factor for all included covariates across these models was confirmed to be < 5, indicating the absence of significant multicollinearity. Restricted cubic spline (RCS) analyses were applied to explore potential nonlinear relationships between LAR values at each time point and 28-day mortality. Subgroup analyses were performed to identify potential effect modifiers in the association between LAR trajectories and both 28- and 90-day mortality among patients with SA-AKI, with results visualized using the *forestploter* R package.

To test the robustness of results, inverse probability of treatment weighting (IPTW) and truncated weight sensitivity analyses were conducted to control for confounding bias [28]. Additionally, a doubly robust (DR) model was constructed using weighted data to evaluate the association between LAR trajectory classes and 28-day mortality, with weighted Kaplan-Meier survival curves plotted for visualization.

For external validation, the GBTM method was used to classify LAR trajectories. Multivariable Cox regression assessed the association between trajectory groups and 28-day mortality, while multivariable logistic regression evaluated their relationship with CRRT. Because APACHE III was not available in the prospective cohort (APACHE II was collected instead; Table S3 in Multimedia Appendix 1), and several primary-cohort covariates (baseline liver disease, exogenous albumin administration, and fluid resuscitation volume) were unavailable, the validation-cohort models were adjusted using routinely available variables (age, SOFA score, AKI stage, and vasopressor use) rather than the primary-cohort adjustment set. This approach assessed the reproducibility of the trajectory classification.

To further quantify the incremental predictive value of the dynamic trajectory model, we systematically compared it against a baseline model using only the single admission LAR (LAR at 0 hour). The improvement in predictive performance for both 28- and 90-day mortality was assessed using Harrell’s concordance index (C-index), continuous net reclassification improvement (NRI), and integrated discrimination improvement (IDI).

To facilitate bedside decision-making, a prognostic nomogram was constructed by integrating the LAR trajectory classes with the core predefined covariates. The predictive performance and clinical applicability of this tool were rigorously evaluated in both the derivation and external validation cohorts. Discrimination was quantified using the C-index, while calibration curves were plotted to assess the agreement between predicted and observed survival probabilities. Furthermore, decision curve analysis and clinical impact curves were generated to evaluate the clinical net benefit and utility at varying threshold probabilities. Finally, patients in the external validation cohort were stratified into distinct risk groups based on their total nomogram scores, and Kaplan-Meier survival curves were used to validate the model’s risk stratification capacity. These predictive modeling and clinical utility analyses were primarily conducted using the *rms* and *rmda* R packages.

All analyses were performed using R software (version 4.5.1; R Foundation for Statistical Computing), and a 2-tailed *P* value <.05 was considered statistically significant.

## Results

### LAR Trajectory Phenotypes in Patients With SA-AKI

**Figure 1.**
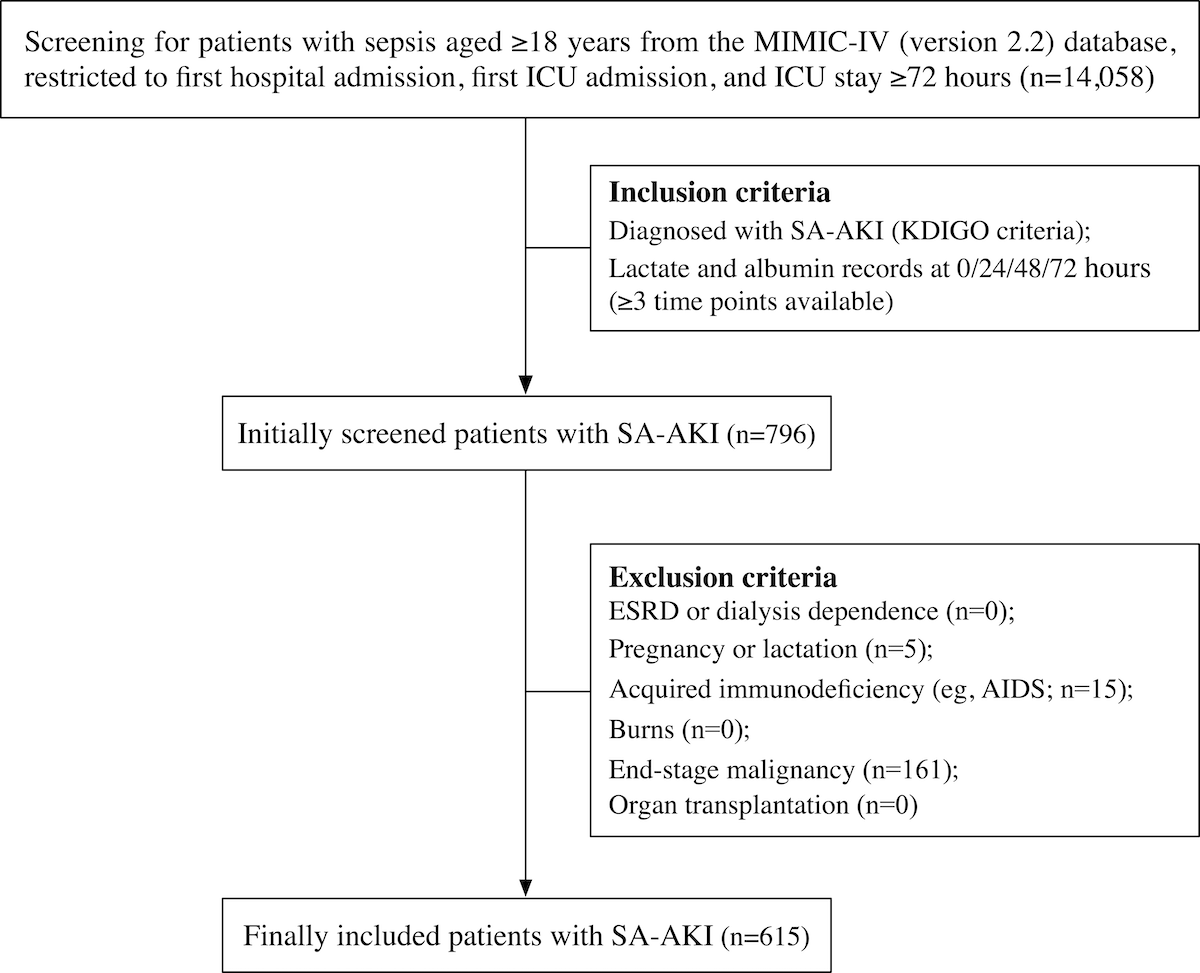
Flowchart of the patient selection process. ESRD: end-stage renal disease; ICU: intensive care unit; KDIGO: Kidney Disease Improving Global Outcomes; MIMIC-IV: Medical Information Mart for Intensive Care IV; SA-AKI: sepsis-associated acute kidney injury.

Figure 1 shows the study flowchart. Of the 14,058 adult patients who were admitted to the ICU for the first time and stayed for more than 72 hours, 615 patients with SA-AKI met the inclusion criteria and were included in the GBTM analysis. To mitigate the influence of extreme values, LAR data were winsorized at the 1st and 99th percentiles prior to modeling (Figure S1 in Multimedia Appendix 1).

**Figure 2.**
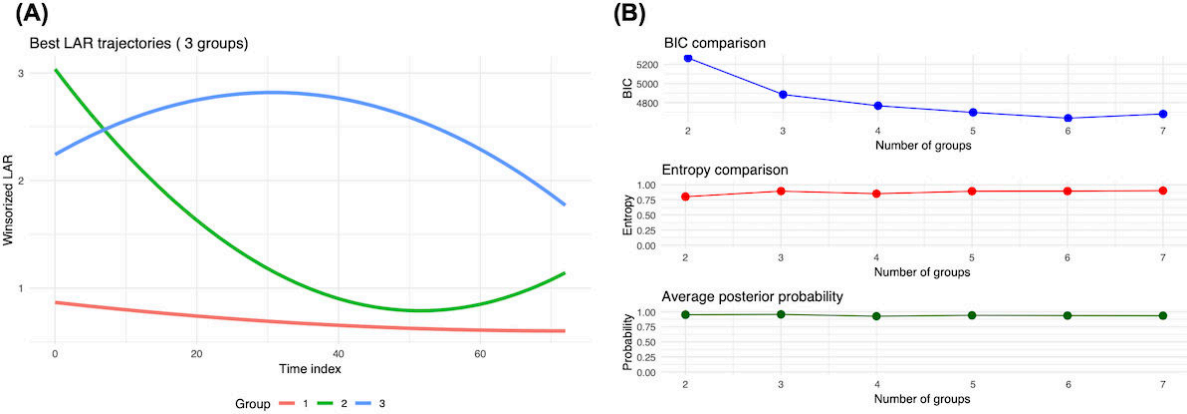
Identification of LAR trajectories. (A) Three classes identified by trajectories of LAR. (B) Comparison of goodness-of-fit indices for the group-based trajectory model of the LAR. BIC: Bayesian information criterion; LAR: lactate-to-albumin ratio.

The model fitting results for 1 to 7 trajectory classes are presented in Table 1 and Figure 2B. A 3-class model was ultimately selected as optimal. Although the 3-class model did not yield the lowest Akaike information criterion or Bayesian information criterion values, it exhibited the highest AvgPostProb, satisfactory entropy, a minimum class proportion >10%, and excellent clinical interpretability. As illustrated in Figure 2A, 3 distinct trajectories of LAR were identified. Trajectory 1 (low-stable type) was characterized by consistently low LAR levels with minimal fluctuation over time. Trajectory 2 (rapid-clearance type) showed markedly elevated baseline LAR levels that decreased rapidly, reflecting an effective and swift clearance of lactate following initial resuscitation, before gradually rising again. Trajectory 3 (delayed-clearance type) exhibited an early increase, indicating an initial failure of lactate clearance and continuous metabolic accumulation, followed by a gradual decline in LAR levels. These findings highlight substantial heterogeneity in LAR evolution among patients with SA-AKI, delineating 3 distinct and clinically meaningful trajectory patterns.

**Table 1.** The group-based trajectory modeling parameters for LAR^a^ trajectory grouping^b^.

Trajectories	Log likelihood	AIC^c^	BIC^d^	Entropy^e^	MinClassSize	AvgPostProb^f^
Class 1	−2993.6	6001.2	6032.03	—^g^	—	—
Class 2	−2595.07606	5214.15212	5267.014862	0.80363679	177	0.950428814
Class 3	−2387.763998	4809.527996	4884.416879	0.896043153	67	0.956239168
Class 4	−2313.309094	4670.618188	4767.533214	0.85254637	44	0.925222932
Class 5	−2262.473016	4578.946033	4697.887201	0.894902026	21	0.94005335
Class 6	−2215.977868	4495.955735	4636.923046	0.896491068	20	0.936190027
Class 7	−2222.224849	4518.449698	4681.443151	0.9041405	16	0.933762705

a LAR: lactate-to-albumin ratio.

b The minimum class size reported here reflects the model-estimated class membership; the analytic group sizes in Table 2 (trajectory 3, n = 68) are based on assignment of each patient to their most likely (modal) trajectory class, which may differ by 1 to 2 patients.

c AIC: Akaike information criterion.

d BIC: Bayesian information criterion.

e Entropy: entropy value.

f AvgPostProb: average posterior probability.

g Not applicable.

**Table 2.** Baseline characteristics of patients grouped according to lactate-to-albumin ratio trajectory.

Variables	Total (n=615)	Trajectory 1 (n=418)	Trajectory 2 (n=129)	Trajectory 3 (n=68)	*P* value
Demographics					
Age (years), median (IQR)	61.00 (49.00-70.00)	60.00 (50.00-71.00)	61.00 (48.00-70.00)	63.50 (51.00-73.00)	.55
Sex, n (%)					.13
Male	376 (61.14)	256 (61.24)	72 (55.81)	48 (70.59)	
Female	239 (38.86)	162 (38.76)	57 (44.19)	20 (29.41)	
Ethnicity, n (%)					.10
Black	62 (10.08)	38 (9.09)	16 (12.40)	8 (11.76)	
White	386 (62.76)	276 (66.03)	68 (52.71)	42 (61.76)	
Other	167 (27.15)	104 (24.88)	45 (34.88)	18 (26.47)	
BMI (kg/m^2^), median (IQR)	28.70 (25.16-33.55)	28.98 (24.90-33.65)	27.10 (25.00-31.53)	29.75 (27.03-33.94)	.03
Vital signs					
Temperature (°C), median (IQR)	36.90 (36.50-37.30)	36.90 (36.60-37.40)	36.70 (36.50-37.10)	36.80 (36.30-37.50)	.007
Heart rate (bpm), mean (SD)	94.39 (18.11)	91.84 (17.91)	97.93 (17.28)	103.35 (17.14)	<.001
Respiratory rate (bpm), median (IQR)	21.00 (18.00-24.00)	20.00 (17.25-24.00)	22.00 (18.00-25.00)	22.00 (19.00-24.00)	.07
SpO₂^a^ (%), median (IQR)	97.00 (96.00-99.00)	98.00 (96.00-99.00)	98.00 (96.00-99.00)	97.00 (95.00-98.00)	.08
SBP^b^ (mm Hg), median (IQR)	109.00 (101.50-118.00)	109.00 (101.00-119.75)	109.00 (102.00-118.00)	108.00 (100.75-115.25)	.52
DBP^c^ (mm Hg), median (IQR)	60.00 (54.00-67.00)	60.00 (54.00-67.00)	61.00 (56.00-68.00)	61.00 (55.75-64.25)	.43
MBP^d^ (mm Hg), median (IQR)	75.00 (70.00-82.00)	75.00 (69.00-82.00)	76.00 (70.00-83.00)	75.00 (70.75-78.25)	.25
Laboratory indicators					
WBC^e^ (×10^9^/L), median (IQR)	9.20 (5.40-14.30)	9.55 (6.30-14.47)	9.00 (5.00-13.60)	6.50 (3.58-10.00)	.003
Platelet (×10^9^/L), median (IQR)	112.00 (57.50-186.00)	135.00 (67.00-206.00)	71.00 (45.00-134.00)	72.00 (44.50-138.25)	<.001
Hemoglobin (g/dL), mean (SD)	9.28 (2.34)	9.64 (2.29)	8.26 (2.33)	9.05 (2.12)	<.001
Hematocrit (%), mean (SD)	28.05 (7.10)	29.11 (6.89)	25.18 (6.99)	27.02 (6.97)	<.001
Lymphocyte (×10^9^/L), median (IQR)	0.81 (0.46-1.36)	0.87 (0.51-1.35)	0.74 (0.38-1.40)	0.54 (0.34-1.33)	.009
Monocyte (×10^9^/L), median (IQR)	0.47 (0.20-0.87)	0.54 (0.25-0.91)	0.34 (0.13-0.76)	0.35 (0.11-0.76)	.001
Neutrophil (×10^9^/L), median (IQR)	9.80 (5.56-14.38)	9.94 (5.95-14.32)	10.06 (5.39-13.96)	7.75 (3.71-15.41)	.22
Glucose (mg/dL), median (IQR)	110.00 (86.00-138.00)	110.50 (90.00-137.00)	108.00 (78.00-137.00)	99.50 (78.50-143.00)	.11
Serum creatinine (mg/dL), median (IQR)	1.20 (0.80-2.10)	1.20 (0.80-2.10)	1.30 (0.90-2.00)	1.35 (0.88-1.80)	.98
Blood urea nitrogen (mg/dL), median (IQR)	24.00 (15.00-41.00)	26.00 (17.00-42.00)	21.00 (13.00-38.00)	24.50 (14.00-36.50)	.04
Anion gap (mmol/L), median (IQR)	14.00 (11.00-17.00)	14.00 (11.00-16.00)	14.00 (12.00-18.00)	15.00 (12.00-20.00)	.001
HCO_3_^−^^f^ (mmol/L), median (IQR)	17.00 (14.00-20.00)	18.00 (16.00-22.00)	15.00 (11.00-18.00)	13.00 (11.00-16.00)	<.001
Na^+g^(mmol/L), median (IQR)	136.00 (132.00-140.00)	136.00 (132.00-140.00)	136.00 (132.00-141.00)	136.00 (133.00-138.00)	.32
K^+h^ (mmol/L), mean (SD)	3.85 (0.66)	3.85 (0.63)	3.83 (0.70)	3.85 (0.78)	.97
Ca^2+i^ (mmol/L), median (IQR)	7.60 (7.00-8.10)	7.70 (7.10-8.20)	7.30 (6.70-8.10)	7.20 (6.80-7.90)	<.001
Cl^−^^j^ (mmol/L), median (IQR)	101.00 (97.00-105.00)	102.00 (97.00-106.00)	102.00 (96.00-105.00)	100.00 (96.75-105.00)	.31
INR^k^ (ratio), median (IQR)	1.40 (1.20-1.80)	1.30 (1.20-1.70)	1.50 (1.30-1.90)	1.50 (1.28-1.80)	.008
PT^l^ (seconds), median (IQR)	15.30 (12.90-19.30)	14.85 (12.70-18.95)	16.60 (14.10-20.70)	16.30 (13.67-19.75)	.007
APTT^m^ (seconds), median (IQR)	31.90 (27.50-38.60)	31.30 (27.40-38.30)	32.30 (28.40-42.00)	33.85 (27.87-40.55)	.11
ALT^n^ (U/L), median (IQR)	44.00 (21.00-156.00)	36.00 (19.00-104.75)	102.00 (28.00-308.00)	45.00 (25.50-216.75)	<.001
ALP^o^ (U/L), median (IQR)	79.00 (51.00-123.00)	78.50 (51.00-121.00)	78.00 (49.00-117.00)	79.50 (51.50-146.00)	.68
AST^p^ (U/L), median (IQR)	78.00 (36.00-251.50)	65.00 (31.00-168.25)	193.00 (59.00-523.00)	92.50 (54.50-311.25)	<.001
TBIL^q^ (mg/dL), median (IQR)	1.30 (0.50-3.00)	1.00 (0.50-2.77)	1.70 (0.70-3.70)	1.60 (0.70-3.75)	<.001
LDH^r^ (U/L), median (IQR)	364.00 (240.00-660.50)	313.00 (231.00-575.00)	472.00 (327.00-1149.00)	523.00 (249.00-981.25)	<.001
ICU^s^ scoring systems					
APACHE III^t^, median (IQR)	85.00 (65.00-106.00)	79.00 (60.25-98.00)	93.00 (73.00-116.00)	102.00 (88.25-123.25)	<.001
SAPS II^u^, mean (SD)	49.99 (14.96)	47.37 (14.38)	54.10 (14.61)	58.28 (14.46)	<.001
SOFA^v^, median (IQR)	5.00 (3.00-7.00)	4.00 (3.00-6.00)	6.00 (4.00-8.00)	6.00 (3.75-8.00)	.002
CCI^w^, median (IQR)	6.00 (4.00-8.00)	6.00 (4.00-8.00)	6.00 (4.00-8.00)	6.00 (4.00-8.00)	.51
Comorbidities					
AKI^x^, n (%)					.004
1	53 (8.62)	42 (10.05)	8 (6.20)	3 (4.41)	
2	182 (29.59)	139 (33.25)	30 (23.26)	13 (19.12)	
3	380 (61.79)	237 (56.70)	91 (70.54)	52 (76.47)	
Hypertension, n (%)	329 (53.50)	223 (53.35)	71 (55.04)	35 (51.47)	.89
Diabetes, n (%)	210 (34.15)	138 (33.01)	54 (41.86)	18 (26.47)	.07
CAD^y^, n (%)	196 (31.87)	142 (33.97)	32 (24.81)	22 (32.35)	.15
HF^z^, n (%)	210 (34.15)	162 (38.76)	29 (22.48)	19 (27.94)	.002
COPD^aa^, n (%)	73 (11.87)	57 (13.64)	12 (9.30)	4 (5.88)	.11
Liver disease, n (%)	204 (33.17)	136 (32.54)	51 (39.53)	17 (25)	.11
Therapeutic interventions, n (%)					
Vasopressor use	246 (40)	134 (32.06)	68 (52.71)	44 (64.71)	<.001
Ventilation	549 (89.27)	371 (88.76)	114 (88.37)	64 (94.12)	.39
CRRT^ab^	177 (28.78)	94 (22.49)	44 (34.11)	39 (57.35)	<.001
Broad-spectrum antibiotics	392 (63.74)	267 (63.88)	77 (59.69)	48 (70.59)	.32
Exogenous albumin administration	212 (34.47)	130 (31.10)	52 (40.31)	30 (44.12)	.03
Fluid resuscitation ≥30 mL/kg	291 (47.32)	176 (42.11)	64 (49.61)	51 (75)	<.001
Outcome measures					
Hospital LOS^ac^ (days), median (IQR)	19.00 (12.00-30.00)	19.00 (12.00-29.00)	19.00 (10.00-28.00)	23.50 (12.00-43.00)	.16
ICU LOS (days), median (IQR)	9.00 (6.00-15.00)	9.00 (6.00-15.00)	8.00 (5.00-13.00)	13.00 (7.75-21.00)	<.001
In-hospital mortality, n (%)	286 (46.50)	184 (44.02)	61 (47.29)	41 (60.29)	.04
28-Day mortality, n (%)	134 (21.79)	74 (17.70)	35 (27.13)	25 (36.76)	<.001
90-Day mortality, n (%)	191 (31.06)	110 (26.32)	47 (36.43)	34 (50)	<.001

a SpO_2_: peripheral oxygen saturation.

b SBP: systolic blood pressure.

c DBP: diastolic blood pressure.

d MBP: mean blood pressure.

e WBC: white blood cell.

f HCO_3_−: bicarbonate.

g Na+: sodium.

h K+: potassium.

i Ca2+: calcium.

j Cl−: chloride.

k INR: international normalized ratio.

l PT: prothrombin time.

m APTT: activated partial thromboplastin time.

n ALT: alanine aminotransferase.

o ALP: alkaline phosphatase.

p AST: aspartate aminotransferase.

q TBIL: total bilirubin.

r LDH: lactate dehydrogenase.

s ICU: intensive care unit.

t APACHE III: Acute Physiology and Chronic Health Evaluation III.

u SAPS II: Simplified Acute Physiology Score II.

v SOFA: Sequential Organ Failure Assessment.

w CCI: Charlson Comorbidity Index.

x AKI: acute kidney injury.

y CAD: coronary artery disease.

z HF: heart failure.

aa COPD: chronic obstructive pulmonary disease.

ab CRRT: continuous renal replacement therapy.

ac LOS: length of stay.

### Baseline Characteristics

The 615 included patients were divided into 3 trajectory groups: trajectory 1 (n=418, 67.97%), trajectory 2 (n=129, 20.97%), and trajectory 3 (n=68, 11.06%). Table 2 compares baseline characteristics of patients across the 3 LAR trajectory types. The 3 groups showed no statistically significant differences in age, sex, race, or the prevalence of preexisting liver disease (*P*>.05). SOFA scores were lowest in trajectory 1, while trajectories 2 and 3 were comparably higher. Clinically, trajectory 3 patients presented with higher BMI and faster heart rates. Laboratory findings revealed lower WBC and lymphocyte counts, HCO_3_^−^, and Ca²^+^, alongside higher AG and lactate dehydrogenase levels. In addition, significantly elevated APACHE III and SAPS II scores in trajectory 3 reflected more pronounced systemic inflammation and organ dysfunction. Furthermore, trajectory 3 had the highest proportion of AKI stage 3 and CRRT initiation, with significantly increased vasopressor use. Consistent with their profound hemodynamic instability, a substantially greater proportion of patients in trajectory 3 required aggressive early fluid resuscitation (≥30 mL/kg; 51/68, 75%; *P*<.001) and exogenous albumin administration (30/68, 44.12%; *P*=.03). Trajectory 2 (rapid-clearance type) patients had lower platelet counts, hemoglobin, hematocrit, and monocyte levels, while PT, INR, ALT, AST, and TBIL were higher, suggesting potential acute hepatic dysfunction despite no significant difference in baseline liver disease history. In contrast, trajectory 1 patients showed relatively higher temperature, WBC count, blood urea nitrogen levels, and HF prevalence, but overall milder clinical severity.

Regarding clinical outcomes, no significant difference was observed in overall hospital length of stay among these 3 groups. However, trajectory 3 patients had longer ICU stays and significantly higher in-hospital, 28-day, and 90-day mortality rates compared with the other 2 groups (all *P*<.05).

### Association Between LAR Trajectories and Outcome Variables

**Figure 3.**
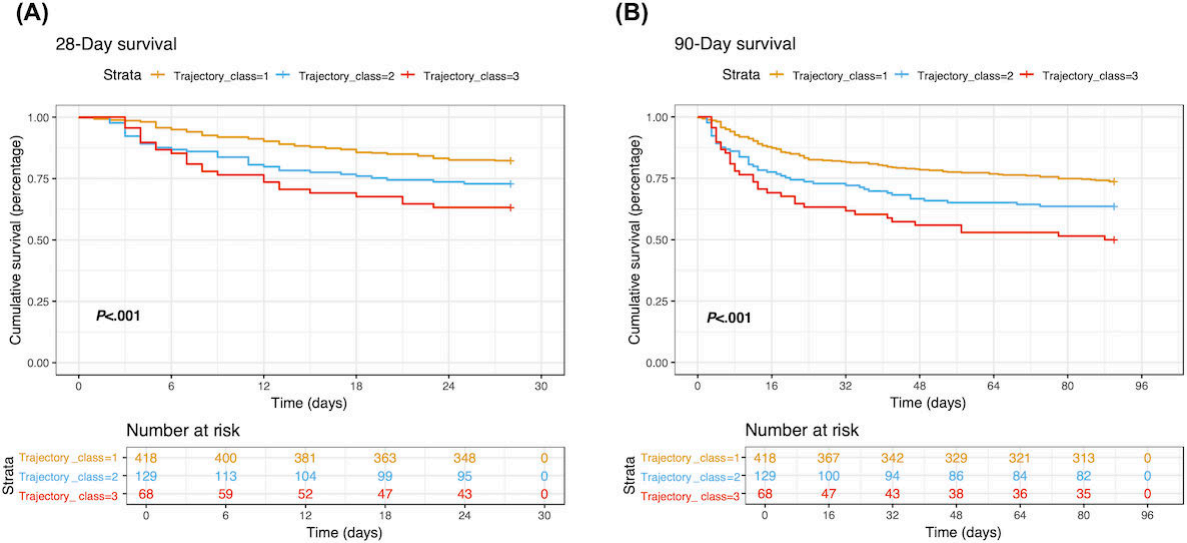
Kaplan-Meier survival analysis for all-cause mortality among each LAR trajectory. Kaplan-Meier curves of (A) 28-day and (B) 90-day all-cause mortality stratified by LAR trajectories. LAR: lactate-to-albumin ratio.

Kaplan-Meier survival curves across LAR trajectory groups are shown in Figure 3. Trajectory 3 exhibited the lowest survival probability among these 3 patient groups (28-day mortality: *P*<.001; 90-day mortality: *P*<.001; Figure 3A and B). Consistent with these survival trends, multivariable logistic regression analysis revealed that trajectory 3 was also associated with a significantly increased risk of in-hospital mortality (OR 1.81, 95% CI 1.07‐3.13; *P*=.03) after adjusting for baseline liver disease, exogenous albumin administration, and fluid resuscitation target (≥30 mL/kg), whereas the risk for trajectory 2 did not differ significantly from the reference group (Table S1 in Multimedia Appendix 1).

**Figure 4.**
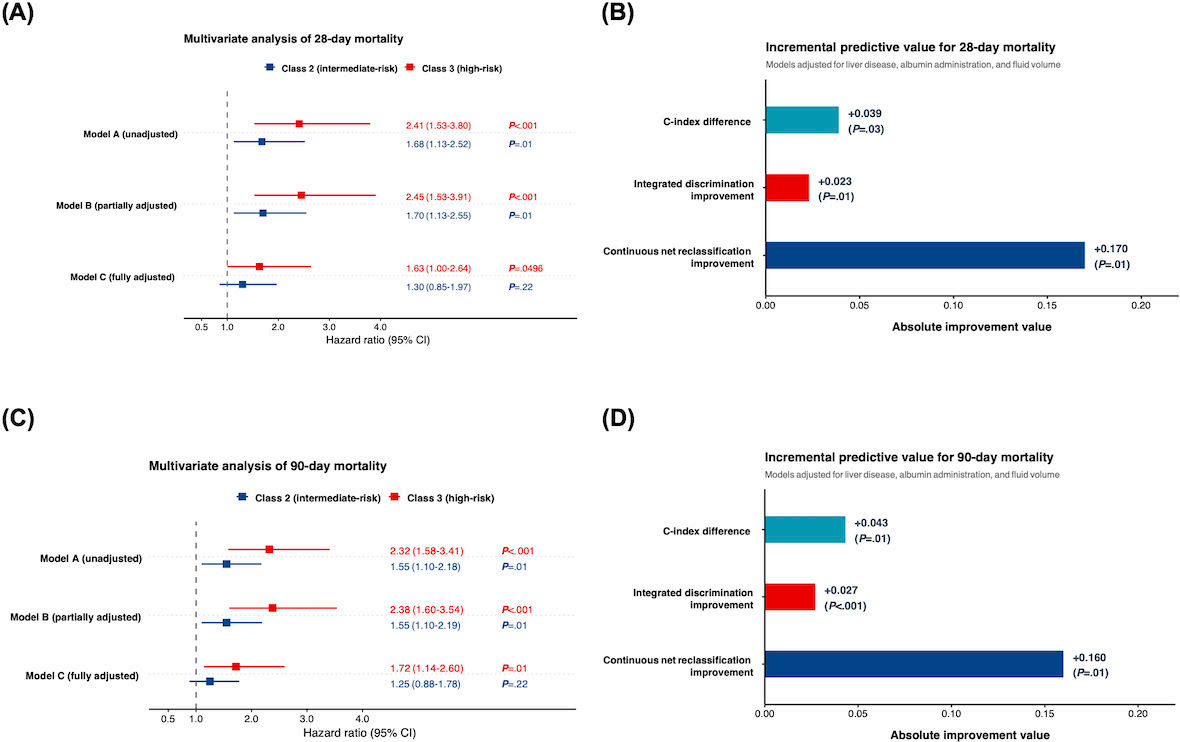
Multivariable regression analyses and incremental predictive value of dynamic LAR trajectories. (A) Multivariable Cox regression analysis of 28-day mortality across 3 progressive models. (B) Incremental predictive value of the trajectory model for 28-day mortality compared to the baseline single LAR model. (C) Multivariable Cox regression analysis of 90-day mortality across 3 progressive models. (D) Incremental predictive value of the trajectory model for 90-day mortality. C-index: concordance index.

To evaluate the independent association between LAR trajectories and clinical outcomes, we constructed 3 progressive multivariable regression models (Table 3 and Figure 4A and C). In the unadjusted analysis (model A) and the partially adjusted analysis (model B, which accounted for baseline liver disease and early resuscitation interventions), both trajectories 2 and 3 remained significantly associated with increased risks of 28-day mortality, 90-day mortality, and CRRT initiation compared to trajectory 1.

However, after further adjusting for overall disease severity and organ dysfunction indicators (model C), the trajectory-outcome associations diverged: trajectory 3, but not trajectory 2, remained an independent risk factor for these adverse outcomes. Specifically, using trajectory 1 as the reference, trajectory 3 was independently associated with a 1.63-fold higher risk of 28-day mortality (hazard ratio [HR] 1.63, 95% CI 1.00‐2.64; *P*=.0496) and a 1.72-fold higher risk of 90-day mortality (HR 1.72, 95% CI 1.14‐2.60; *P*=.01). Conversely, the mortality risk for trajectory 2 (rapid-clearance type) did not differ significantly from trajectory 1 in the fully adjusted model (28-day: HR 1.30, 95% CI 0.85‐1.97; *P*=.22; 90-day: HR 1.25, 95% CI 0.88‐1.78; *P*=.22). Consistent findings were observed for the CRRT initiation; multivariable logistic regression (model C) demonstrated that trajectory 3 patients had a substantially higher risk of requiring CRRT (odds ratio [OR] 3.40, 95% CI 1.64‐7.15; *P*=.001), whereas trajectory 2 was not independently associated with increased CRRT use (OR 0.92, 95% CI 0.53‐1.58; *P*=.77).

**Table 3.** Multivariable regression analysis of the association between lactate-to-albumin ratio trajectories and 28-day mortality, 90-day mortality, and CRRT^a^ in patients with sepsis-associated acute kidney injury.

Model and trajectory class	28-Day mortality		90-Day mortality		CRRT initiation	
	HR^b^ (95% CI)	*P* value	HR (95% CI)	*P* value	OR^c^ (95% CI)	*P* value
Model A: unadjusted						
Class 1	Reference	—^d^	Reference	—	Reference	—
Class 2	1.68 (1.13‐2.52)	.01	1.55 (1.10‐2.18)	.01	1.78 (1.16‐2.74)	.008
Class 3	2.41 (1.53‐3.80)	<.001	2.32 (1.58‐3.41)	<.001	4.64 (2.73‐7.95)	<.001
Model B: partially adjusted^e^						
Class 1	Reference	—	Reference	—	Reference	—
Class 2	1.70 (1.13‐2.55)	.01	1.55 (1.10‐2.19)	.01	1.79 (1.14‐2.77)	.01
Class 3	2.45 (1.53‐3.91)	<.001	2.38 (1.60‐3.54)	<.001	5.81 (3.31‐10.38)	<.001
Model C: fully adjusted						
Class 1	Reference	—	Reference	—	Reference	—
Class 2	1.30 (0.85‐1.97)	.22	1.25 (0.88‐1.78)	.22	0.92 (0.53‐1.58)	.77
Class 3	1.63 (1.00‐2.64)	.0496^f^	1.72 (1.14‐2.60)	.01^f^	3.40 (1.64‐7.15)	.001^g^

a CRRT: continuous renal replacement therapy.

b HR: hazard ratio.

c OR: odds ratio.

d Not applicable.

e Adjusted for baseline liver disease, exogenous albumin administration, and fluid resuscitation target (≥30 mL/kg).

f Adjusted for variables in model B, plus Acute Physiology and Chronic Health Evaluation III score, acute kidney injury stage, heart failure, and vasopressor use.

g Adjusted for variables in model B, plus acute kidney injury stage, Sequential Organ Failure Assessment score, vasopressor use, and anion gap.

### RCS and Subgroup Analysis

RCS models were used to explore the nonlinear association between LAR and 28-day mortality in patients with SA-AKI (Figure S2 in Multimedia Appendix 1). A significant nonlinear relationship was observed at all 4 time points (0, 24, 48, and 72 hours; all *P*<.05). Notably, the mortality risk gradients steepened progressively at later time intervals (24‐72 hours) compared with the baseline (0 hours). These temporal dynamics demonstrate that a persistently elevated LAR confers a disproportionately higher mortality hazard, underscoring the prognostic superiority of serial assessments over a single admission value.

**Figure 5.**
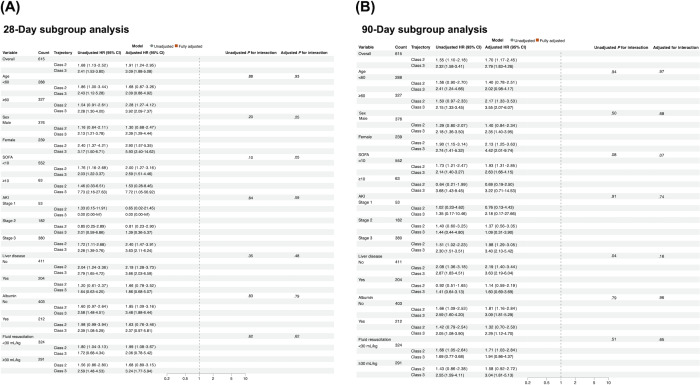
Subgroup analyses of the associations between LAR trajectories and mortality. (A) Forest plot of subgroup analysis for 28-day mortality. (B) Forest plot of subgroup analysis for 90-day mortality. Both unadjusted and fully adjusted HRs are presented, with *P* values for interaction indicating no significant effect modification across strata. AKI: acute kidney injury; HR: hazard ratio; LAR: lactate-to-albumin ratio; SOFA: Sequential Organ Failure Assessment.

Figure 5 presents the subgroup analyses evaluating the association between LAR trajectory classes and 28-day (Figure 5A) as well as 90-day mortality (Figure 5B). Patients were stratified by clinically relevant baseline characteristics and early interventions, including age, sex, SOFA score, AKI stage, liver disease, exogenous albumin administration, and fluid resuscitation volume (≥30 mL/kg). No significant interactions were detected across any of the predefined strata (all *P* for interaction >.05), demonstrating that the prognostic impact of the LAR trajectories was consistent across diverse clinical subpopulations. Crucially, in alignment with our primary multivariable analysis, the fully adjusted forest plots confirmed that trajectory 3 (delayed-clearance type) maintained a consistent and independent association with higher mortality risk across most subgroups. Conversely, the mortality risk associated with trajectory 2 was largely attenuated and lost statistical significance upon full adjustment within these strata.

### Sensitivity Analyses

**Figure 6.**
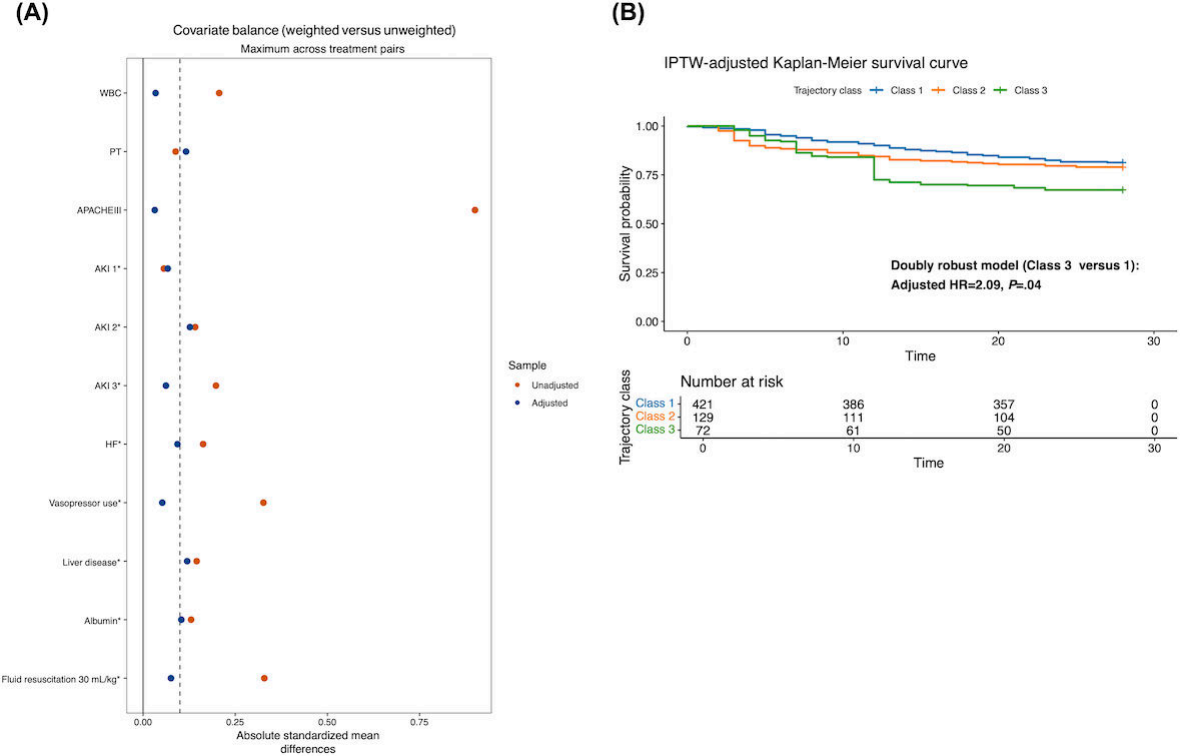
Sensitivity analyses evaluating the robustness of LAR trajectories using IPTW and a doubly robust model. (A) Covariate balance before (unadjusted) and after (adjusted) IPTW. The dashed line indicates an absolute standardized mean difference of 0.1, showing excellent balance across covariates. (B) IPTW-adjusted Kaplan-Meier survival curves and the adjusted HR derived from the doubly robust model for 28-day mortality. AKI: acute kidney injury; APACHE III: Acute Physiology and Chronic Health Evaluation III; HF: heart failure; HR: hazard ratio; IPTW: inverse probability of treatment weighting; LAR: lactate-to-albumin ratio; PT: prothrombin time; WBC: white blood cell.

After applying IPTW based on multinomial propensity scores, the absolute standardized mean differences for all covariates were substantially reduced to <0.1 (indicated by the dashed line), demonstrating excellent covariate balance and successful mitigation of baseline confounding across the LAR trajectory groups (Figure 6A).

As shown in Table 4, the weighted Cox regression (untruncated) revealed that the mortality risk for trajectory 2 did not differ significantly from trajectory 1 (HR 1.19, 95% CI 0.75‐1.88; *P*=.45), while trajectory 3 exhibited a trend toward higher risk (HR 1.95, 95% CI 0.93‐4.09; *P*=.08). After trimming extreme weights at the 1st and 99th percentiles to reduce the influence of extreme values, the results remained consistent, yielding HRs of 1.20 and 1.79 for trajectories 2 and 3, respectively.

To rigorously account for any residual confounding, a DR Cox model was used (IPTW+covariate adjustment). In this DR model, trajectory 3 remained significantly and independently associated with higher 28-day mortality (HR 2.09, 95% CI 1.03‐4.24; *P*=.04), whereas trajectory 2 maintained no significant association (HR 1.24, 95% CI 0.79‐1.95; *P*=.34). These findings are visually corroborated by the IPTW-adjusted Kaplan-Meier survival curves (Figure 6B), where trajectory 3 continues to exhibit the lowest survival probability. However, in the IPTW-weighted analyses (both untruncated and truncated), the association for trajectory 3 was attenuated and did not reach statistical significance (*P*=.08 and *P*=.07, respectively), and statistical significance was only observed in the DR model. Given that the primary fully adjusted estimate was also borderline (HR 1.63, 95% CI 1.00‐2.64), these results indicate that the estimated effect for trajectory 3 is sensitive to the analytical framework. Accordingly, these findings should be interpreted as supportive but not conclusive evidence of an independent association.

**Table 4.** Association between lactate-to-albumin ratio trajectory classes and 28-day mortality after IPTW^a^ and double robust adjustment.

Model and comparison vs trajectory 1	HR^b^ (95% CI)	*P* value	Concordance	Weighted method
IPTW-Cox (untruncated)			0.553	Stabilized weights
Trajectory 2	1.19 (0.75‐1.88)	.45		
Trajectory 3	1.95 (0.93‐4.09)	.08		
IPTW-Cox (truncated)			0.545	Truncated weights (1st-99th percentile)
Trajectory 2	1.20 (0.76‐1.88)	.44		
Trajectory 3	1.79 (0.95‐3.39)	.07		
Double robust Cox model			0.685	IPTW+covariate adjustment
Trajectory 2	1.24 (0.79‐1.95)	.34		
Trajectory 3	2.09 (1.03‐4.24)	.04		

a IPTW: inverse probability of treatment weighting.

b HR: hazard ratio.

### External Validation Results

**Figure 7.**
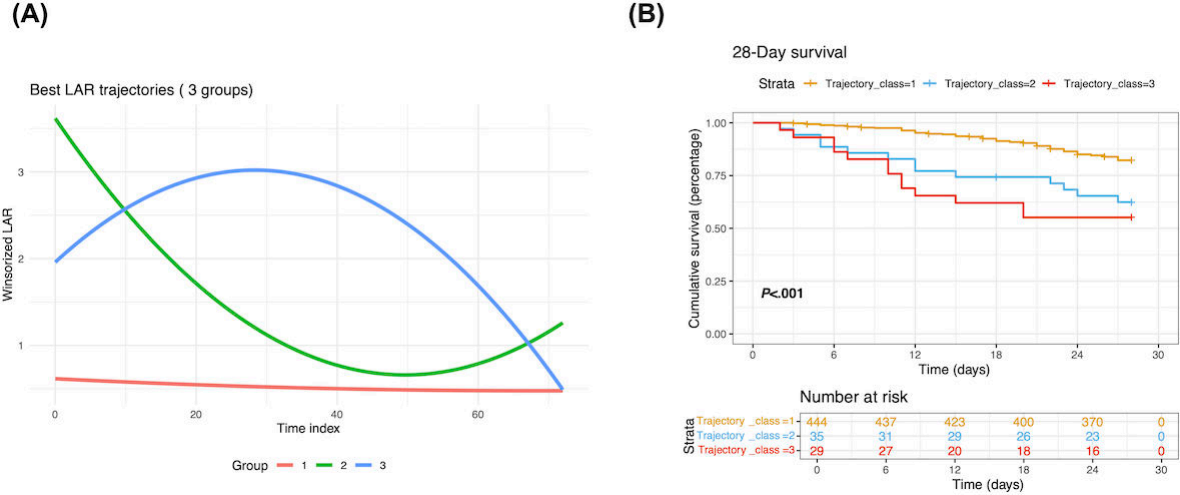
LAR trajectories and Kaplan-Meier survival analysis on the external validation set. (A) Three classes identified by LAR trajectories on the external validation set, and (B) Kaplan-Meier survival analysis for 28-day all-cause mortality among each LAR trajectory on the external validation set. LAR: lactate-to-albumin ratio.

In the external validation cohort (n=508), 3 distinct LAR trajectory patterns were identified, with time-course patterns consistent with those observed in the MIMIC-IV cohort. These included trajectory 1 (low-stable type), trajectory 2 (rapid-clearance type), and trajectory 3 (delayed-clearance type; Figure 7A and Table S2 in Multimedia Appendix 1). Patients in trajectory 3 exhibited poorer prognosis, with a significantly higher 28-day mortality rate (Figure 7B). Comparison of baseline characteristics (Table S3 in Multimedia Appendix 1) revealed that trajectory 3 patients exhibited more pronounced inflammation, more severe coagulopathy, greater multiorgan dysfunction, and higher rates of CRRT initiation, in line with the findings from the MIMIC-IV cohort. Multivariable regression analyses were conducted on the validation set to further assess the relationship between LAR trajectories and clinical outcomes (Table 5). For 28-day mortality, multivariable Cox regression analysis confirmed that trajectory 3 (delayed-clearance type) carried the highest mortality risk, demonstrating a 2.86-fold increase compared with trajectory 1 (HR 2.86, 95% CI 1.57‐5.22; *P*<.001). In this validation cohort, trajectory 2 was also significantly associated with an elevated risk of 28-day mortality (HR 2.49, 95% CI 1.35‐4.59; *P*=.003). Cross-cohort analyses revealed that trajectory 2 patients from the validation cohort were more critically ill at baseline than their MIMIC-IV counterparts, with higher SOFA scores and more frequent vasopressor use (Table S4 in Multimedia Appendix 1). Conversely, multivariable logistic regression analysis revealed that the LAR trajectory type did not independently predict the initiation of CRRT in the validation set. While patients in trajectory 3 (OR 1.89, 95% CI 0.79‐4.76; *P*=.16) and trajectory 2 (OR 1.62, 95% CI 0.71‐3.68; *P*=.25) showed a clinical tendency toward requiring CRRT, neither association reached statistical significance.

**Table 5.** Multivariable regression analysis of the association between lactate-to-albumin ratio trajectories with 28-day mortality and CRRT^a^ in patients with sepsis-associated acute kidney injury on the validation set.

Variables	28-Day mortality (Cox regression)		CRRT^a^ initiation (logistic regression)	
	HR^b^ (95% CI)	*P* value	OR^c^ (95% CI)	*P* value
Trajectory class				
Class 1	1.00 (reference)	—^d^	1.00 (reference)	—
Class 2	2.49 (1.35‐4.59)	.003	1.62 (0.71‐3.68)	.25
Class 3	2.86 (1.57‐5.22)	<.001	1.89 (0.79‐4.76)	.16
Age	1.01 (1.00‐1.03)	.03	0.99 (0.98‐1.01)	.23
SOFA^e^ score	1.11 (1.05‐1.17)	<.001	1.16 (1.10‐1.24)	<.001
Acute kidney injury stage				
Stage 1	1.00 (reference)	—	1.00 (reference)	—
Stage 2	1.09 (0.63‐1.89)	.75	1.14 (0.66‐1.94)	.63
Stage 3	1.53 (0.98‐2.40)	.06	4.33 (2.70‐7.04)	<.001
Vasopressor use	1.30 (0.69‐2.47)	.42	3.34 (1.72‐6.83)	<.001

a CRRT: continuous renal replacement therapy.

b HR: hazard ratio.

c OR: odds ratio.

d Not applicable.

e SOFA: Sequential Organ Failure Assessment.

### Incremental Predictive Value and External Validation of the Clinical Nomogram

To evaluate whether dynamic LAR trajectories offer superior prognostic utility compared with a single baseline measurement, we assessed their incremental predictive value using C-index, continuous NRI, and IDI (Table 6 and Figure 4B and D). For 28-day mortality, integrating the LAR trajectory class into the baseline model significantly improved its discriminative ability (C-index increased from 0.579 to 0.618; *P*=.03). This enhancement was further supported by a continuous NRI of 0.170 (*P*=.01) and an IDI of 0.023 (*P*=.01), indicating that the trajectory model correctly reclassified a substantial proportion of patients. Consistent improvements in predictive accuracy were similarly observed for 90-day mortality (Table 6). These findings strongly suggest that the temporal evolution of LAR provides critical prognostic information beyond what a single admission value can offer.

**Table 6.** Incremental predictive value of dynamic LAR^a^ trajectories compared to single baseline LAR.

Predictive model^b^	C-index^c^	*P* value^d^	Continuous NRI^e^ (95% CI)	*P* value	IDI^f^ (95% CI)	*P* value
28-Day mortality						
Baseline model (single LAR)	0.579	Reference	Reference	—^g^	Reference	—
Trajectory model (LAR trajectories)	0.618	.03	0.170 (0.047‐0.249)	.01	0.023 (0.004‐0.053)	.01
90-Day mortality						
Baseline model (single LAR)	0.559	Reference	Reference	—	Reference	—
Trajectory model (LAR trajectories)	0.602	.006	0.160 (0.056‐0.225)	.007	0.027 (0.006‐0.056)	<.001

a LAR: lactate-to-albumin ratio.

b Both the baseline model and the trajectory model were strictly matched and adjusted for liver disease, exogenous albumin administration, and fluid resuscitation target (≥30 mL/kg).

c C-index: concordance index.

d *P* value for the comparison of C-indices was calculated using the Noether method.

e NRI: net reclassification improvement.

f IDI: integrated discrimination improvement.

g Not applicable.

**Figure 8.**
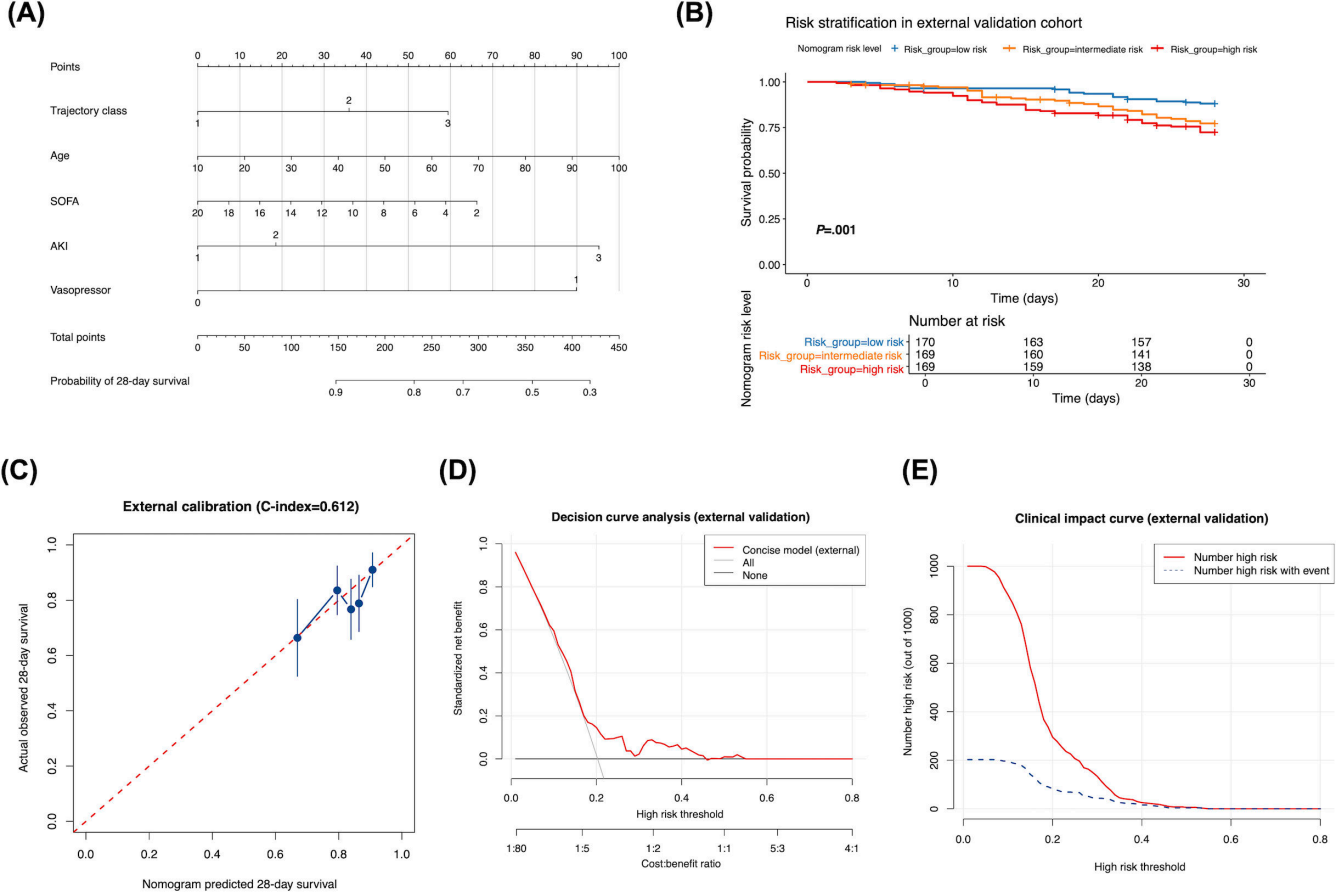
Construction and external validation of the trajectory-integrated clinical nomogram. (A) Nomogram incorporating LAR trajectory class and clinical variables for predicting 28-day survival probability in patients with SA-AKI. (B) Kaplan-Meier survival curves demonstrating effective risk stratification (low, intermediate, and high risk) based on nomogram scores in the external validation cohort. (C) Calibration curve of the nomogram in the external validation cohort. (D) Decision curve analysis demonstrating the clinical net benefit of the concise model in the external validation cohort. (E) Clinical impact curve evaluating the practical utility of the nomogram in the external validation cohort. AKI: acute kidney injury; C-index: concordance index.

To translate these findings into a practical bedside tool, we constructed a prognostic nomogram for predicting 28-day survival. As illustrated in Figure 8A, the nomogram incorporates the LAR trajectory class alongside standard clinical predictors: age, SOFA score, AKI stage, and vasopressor use. The clinical reliability and generalizability of this model were subsequently evaluated in an independent multicenter validation cohort from tertiary hospitals in Beijing. Additionally, a web-based dynamic prognostic calculator with a user-friendly interface is available to facilitate bedside risk assessment (Figure S3 in Multimedia Appendix 1).

Applying the nomogram to the external validation cohort allowed for effective risk stratification. Patients were stratified into low-, intermediate-, and high-risk groups, with Kaplan-Meier analysis showing statistically detectable separation in survival probabilities among the 3 strata (*P*=.001; Figure 8B). The nomogram exhibited satisfactory discrimination in the external cohort with a C-index of 0.612, and the calibration curve showed favorable concordance between the nomogram-predicted and actual observed 28-day survival probabilities (Figure 8C). Furthermore, decision curve analysis confirmed that the concise model yielded a positive standardized net benefit across a wide range of high-risk thresholds, outperforming the “treat-all” and “treat-none” strategies (Figure 8D). The clinical impact curve additionally suggested the model’s practical utility in accurately identifying high-risk individuals in a real-world clinical setting, primarily functioning as a supplementary tool for bedside risk stratification rather than a standalone predictive model (Figure 8E).

## Discussion

### Principal Findings

In this derivation and multicenter trajectory-based study of patients with SA-AKI, we identified 3 distinct early LAR trajectories and demonstrated that the delayed-clearance trajectory (trajectory 3) was independently associated with 28- and 90-day mortality. Importantly, dynamic LAR trajectories provided incremental prognostic value beyond single baseline LAR, and the trajectory-integrated nomogram showed reproducible risk stratification in an external validation cohort. These findings suggest that the temporal evolution of LAR, rather than its baseline value alone, may better capture the dynamic pathophysiology of SA-AKI. Although the overall trajectory shapes identified in the external validation cohort were analogous to those in the MIMIC-IV cohort, substantial differences in trajectory distribution were evident. Specifically, the proportion of patients assigned to the low-stable trajectory increased from 67.97% in the MIMIC cohort to 87.4% in the validation cohort, whereas the rapid-clearance and delayed-clearance trajectories became less frequent (6.9% and 5.7%, respectively). These findings indicate that while the trajectory framework itself was reproducible, the prevalence of individual trajectory classes varied substantially across cohorts. Several factors may contribute to this distributional shift, including differences in patient case-mix, health care system structures, ICU admission thresholds, local resuscitation practices, and the timing of ICU admission relative to sepsis onset.

### Pathophysiological Insights Into LAR Trajectories

Multiple studies have demonstrated that LAR is closely associated with mortality in SA-AKI and may serve as an emerging composite biomarker with significant value in assessing disease severity and prognosis in critically ill patients [17,29,30]. However, LAR changes with SA-AKI disease progression and may serve as a “dynamic marker” of disease evolution. A study from Athens measured LAR both at sepsis diagnosis and 1 week later, demonstrating that LAR was associated with sepsis severity and mortality in both early and late disease stages [19]. Research by Shadvar et al [31] showed that LAR at 6 hours after septic shock onset had high predictive value for mortality, with the area under the receiver operating characteristic curve reaching 91.7%. Consequently, dynamic monitoring of LAR is crucial for assessing septic shock. Nevertheless, the longitudinal trajectory of LAR during the course of SA-AKI and its association with clinical outcomes has not been fully elucidated. Thus, systematically evaluating the dynamic changes of LAR holds significant clinical value for predicting the prognosis of SA-AKI. Furthermore, existing research indicates that most AKI cases resolve within 72 hours, with persistent severe AKI defined as progression from AKI stage 2‐3 (KDIGO classification) to stage 3 persisting ≥72 hours [32], highlighting the importance of early SA-AKI management. Therefore, it is reasonable that this study selected the 72-hour time window after SA-AKI onset to analyze LAR dynamic changes.

In this study, we identified 3 LAR dynamic trajectory classes: trajectory 1 (low-stable type), trajectory 2 (rapid-clearance type), and trajectory 3 (delayed-clearance type). Among these, trajectory 1 had the lowest overall critical illness scores and best outcomes, representing a “low-risk stable trajectory” group with consistently low LAR values (<1). Trajectory 3 had the most severe disease course, complicated by metabolic acidosis, renal function deterioration, and circulatory deterioration, with markedly higher critical illness scores, more pronounced multiorgan dysfunction, the highest subsequent CRRT initiation, and the highest 28- and 90-day mortality rates (*P*<.05). Furthermore, Kaplan-Meier survival analysis and multivariable Cox regression suggested an association between trajectory 3 and 28-day mortality in patients with SA-AKI. RCS and subgroup analyses were directionally consistent. However, in IPTW-weighted models, the association was attenuated and no longer statistically significant; significance was only observed under DR adjustment. Consequently, this finding appears sensitive to the analytical approach and should be interpreted with caution. Trajectory 2 presented with markedly elevated baseline LAR, initially mimicking a critically ill state, but experienced a rapid decline. This pattern may reflect preserved physiologic reserve, quick restoration of tissue perfusion, and a favorable biochemical response to early resuscitation. In contrast, trajectory 3 showed a more prolonged metabolic disturbance. Consistent with established pathophysiological mechanisms of persistent hyperlactatemia and severe systemic inflammation in sepsis [33], this delayed-clearance phenotype likely reflects a “refractory” state driven by underlying microcirculatory shunting, mitochondrial dysfunction, and impaired hepatic clearance. These patients may initially appear hemodynamically stabilized; yet, the delayed clearance signals an ongoing, insidious cellular hypoxia that ultimately drives progressive organ damage. The successful reproduction of these trajectory models in the multicenter validation cohort supports their external validity, despite cohort-level variations in class distribution and effect sizes. These results indicate that the dynamic trajectory of LAR may serve as a prognostic indicator in patients with SA-AKI, potentially facilitating early identification of high-risk individuals and supporting the development of individualized therapeutic strategies to slow disease progression and improve clinical outcomes.

### Clinical Actionability and Bedside Translation

Prior studies have reported markedly different optimal cutoff values for LAR. For example, Bou Chebl et al [34] identified thresholds of 1.22 for sepsis and 1.47 for septic shock in an emergency department population, whereas Zhu et al [35] found that LAR ≥1.49 independently predicted in-hospital and ICU mortality among critically ill patients with AKI. In another study, Wang and Yu [30] determined a cutoff value of LAR ≥0.95 associated with increased mortality in SA-AKI. These inconsistencies are likely attributable to differences in study populations, sample sizes, and timing of LAR measurement. Our study overcomes the inherent limitations of these static cutoffs by demonstrating the substantial incremental predictive value of dynamic trajectories. Integrating the trajectory class significantly improved model discrimination (C-index) and reclassification (NRI and IDI) compared to a single baseline LAR. Although statistically significant, the improvement in model discrimination was numerically modest (absolute C-index of approximately 0.61). This performance is consistent with existing ICU prognostic scores, which typically report C-indices between 0.60 and 0.70 [36,37]. Given the multifactorial nature of SA-AKI, no single biomarker can fully capture outcome heterogeneity. In this context, LAR trajectories may provide additional phenotypic information that helps refine bedside risk stratification and identify patients at higher risk of adverse outcomes when interpreted alongside established clinical scores. Furthermore, our RCS analysis highlighted a potential therapeutic time window. Because mortality risk curves steepened progressively at 24 and 48 hours compared to the 0-hour baseline, we suggest that the 24- to 48-hour window may represent an important period for clinical reassessment. If LAR fails to decline during this time, it warrants a thorough evaluation of hemodynamic adequacy, tissue perfusion, metabolic status, and evolving organ dysfunction, rather than relying on apparent macrocirculatory stabilization alone. While formal trajectory classification requires 72 hours of data, early LAR dynamics may provide clinically relevant information before trajectory assignment is possible. A stagnant or rising LAR during the first 24 to 48 hours may signal inadequate physiological recovery and warrants close reassessment, as these patients may ultimately be classified into the delayed-clearance trajectory. Because the externally validated nomogram relies on the complete 72-hour LAR profile, it functions as a day-3 prognostic tool rather than a real-time resuscitation guide. It is designed to reflect the cumulative physiological response to early treatment and inform subsequent risk stratification.

Trajectory 2 (rapid-clearance type) was particularly noteworthy in this study. Patients in this group exhibited marked hepatic dysfunction, evidenced by significant elevations in PT, INR, ALT, AST, and TBIL, suggesting concurrent or evolving liver injury. From a pathophysiological standpoint, hepatic dysfunction may exert a dual influence on LAR, as impaired hepatic metabolism and clearance of lactate lead to lactate accumulation [38], while reduced albumin synthesis reflects both malnutrition and hepatic synthetic failure [39]. Consequently, LAR levels in this group may be artifactually elevated. These findings highlight the importance of interpreting dynamic LAR changes in conjunction with hepatic function, given the potential bias introduced by liver injury. Notably, previous research has shown that LAR retains prognostic value for mortality even among patients with sepsis and cirrhosis [40], underscoring its robustness and clinical applicability under complex pathophysiologic conditions. Crucially, after rigorous multivariable adjustment for disease severity, baseline liver disease, and early resuscitation interventions (such as fluid volume ≥30 mL/kg and exogenous albumin), the mortality risk associated with trajectory 2 was attenuated and lost statistical significance. This suggests that the initially elevated risk in trajectory 2 may be largely mitigated by appropriate standard care. Although this interpretation may account for the findings observed in the MIMIC cohort, a different pattern was observed in the external validation cohort. After full adjustment, trajectory 2 remained significantly associated with 28-day mortality in the validation cohort, whereas no independent association was observed in the derivation cohort. Compared with patients assigned to trajectory 2 in the MIMIC-IV cohort, those in the validation cohort were older and had higher illness severity at baseline, including higher SOFA scores and more frequent vasopressor use (Table S4 in Multimedia Appendix 1). These differences suggest that patients sharing a similar LAR trajectory pattern may represent distinct clinical phenotypes across health care settings. Furthermore, exogenous albumin administration was not available in the prospective cohort, which may partly account for differences in the observed performance of trajectory 2 between cohorts. While the trajectory framework itself was reproducible, the apparent prognostic separation between classes is likely influenced by case-mix differences and variation in covariate availability. In contrast, trajectory 3 showed an association with mortality in the primary and DR models, although results were not consistent across IPTW-weighted analyses. Given the marginal significance of the primary estimate (HR 1.63, 95% CI 1.00‐2.64) and attenuation in IPTW models, the stability of this association should be interpreted with caution. Nevertheless, the directionality of the effect remained consistent across models, suggesting a persistent signal of increased mortality risk in the delayed-clearance phenotype, despite standard fluid or albumin resuscitation. These findings suggest that conventional interventions may be insufficient to fully overcome the profound underlying pathophysiological derangements in this specific subgroup, underscoring the need for vigilant monitoring and the exploration of individualized or intensified therapeutic strategies.

In this study, the CRRT initiation was defined as a secondary end point. In the MIMIC cohort, multivariable logistic regression analysis showed that only trajectory 3 was independently associated with CRRT initiation, whereas trajectory 2 was not. Patients in trajectory 3 exhibited markedly higher AG, lower HCO_3_^−^ levels, a greater proportion of KDIGO stage 3 AKI, and higher vasopressor requirements, indicating that they were more likely to meet CRRT initiation criteria. In contrast, patients in trajectory 2 tended to present with hepatic dysfunction and a relatively lower proportion of KDIGO stage 3 AKI than those in trajectory 3. For them, the clinical benefits of CRRT appear to be limited. However, in our external validation cohort, this association did not reach statistical significance. These findings indicate that the optimal timing of CRRT initiation in SA-AKI remains uncertain [41] and that LAR trajectories alone may be insufficient to guide decisions regarding CRRT. Clinical assessment, metabolic derangements, and multiorgan function should be jointly considered when determining CRRT initiation.

### Implications for Future Clinical Trials

Beyond guiding individualized bedside management, identification of the delayed-clearance LAR trajectory may also have important implications for future clinical trial design. Previous pharmacologic trials in SA-AKI have frequently failed, in part because of the substantial clinical heterogeneity of septic populations. Enrolling low-risk patients who are likely to recover with standard care alone, such as those in trajectory 1 or trajectory 2, may dilute the true treatment effect of investigational interventions. Prognostic enrichment [42] based on dynamic LAR trajectories could allow future randomized controlled trials to selectively enroll patients with a high-risk delayed-clearance phenotype who respond poorly to conventional treatment. Such a targeted enrollment strategy may improve statistical power and more accurately define the clinical value of novel metabolic-modulating therapies or extracorporeal organ support strategies.

### Strengths and Limitations

The major strengths of this study include the use of longitudinal trajectory modeling, comprehensive sensitivity analyses, comparison with single baseline LAR, and consistent external validation in a prospective multicenter cohort. Nevertheless, this study has several limitations. First, as a single-center study based on the MIMIC database, while validated by a multicenter prospective cohort, the findings may not be generalizable to community hospitals. Second, due to the lack of precise timestamps, CRRT could not be treated as a time-varying exposure. In patients who initiated CRRT early, subsequent LAR measurements may have reflected, in part, machine-driven solute clearance rather than purely physiological lactate handling. Consequently, the observed trajectory patterns may have been influenced by treatment effects in addition to the underlying disease process. Furthermore, because trajectory classification requires 72 hours of data, CRRT may be initiated before this window is completed, introducing temporal ambiguity into the secondary outcome analysis. This analysis should therefore be interpreted as an observational association rather than a causal effect. Third, in the multicenter external validation cohort (collected between June 2023 and October 2025), routine COVID-19 nucleic acid testing was no longer widely implemented in the postpandemic era. Consequently, specific data on COVID-19 infection status were largely unmeasured. Despite this potential unmeasured confounding regarding viral etiology, the trajectory model and nomogram retained modest discrimination (C-index 0.612) while showing acceptable calibration and positive net clinical benefit in this cohort, suggesting its generalizability across different clinical settings. Fourth, although multiple adjustment strategies such as multivariable modeling, IPTW, and DR estimation were used to minimize confounding bias, residual confounders may still exist. Specifically, the external validation cohort lacked data on baseline liver disease, exogenous albumin administration, and fluid resuscitation volume, resulting in a different adjustment set between cohorts. The absence of exogenous albumin data is particularly relevant, as it acts as an unmeasured confounder that may influence the observed prognostic performance of trajectory 2. This external validation represents a pragmatic assessment under real-world data constraints rather than a fully harmonized replication. Finally, the ascertainment of 90-day mortality in the primary MIMIC-IV cohort relied on hospital records and linked mortality data available within the database. Patients discharged alive without subsequent recorded death events were assumed to be alive at 90 days. As MIMIC-IV may not capture all out-of-hospital deaths, this approach could introduce ascertainment bias and potentially underestimate long-term mortality. Therefore, the 90-day mortality findings should be interpreted with appropriate caution. Addressing these limitations will help to clarify the clinical implications of LAR trajectories for prognostic assessment in patients with SA-AKI.

### Conclusions

This study identified distinct dynamic trajectories of the LAR in patients with SA-AKI. The findings demonstrated that temporal LAR trajectories were associated with both 28- and 90-day mortality, with the “delayed-clearance” pattern conferring the highest risk. Furthermore, this trajectory was associated with CRRT initiation in the primary cohort. Dynamic LAR trajectories may serve as consistent composite biomarkers for characterizing disease progression and prognosis in SA-AKI, providing significant incremental predictive value over static baseline measurements. Failure of LAR clearance within the early 24- to 48-hour window may help identify patients who warrant closer monitoring and reassessment of perfusion, metabolic status, renal dysfunction, and overall therapeutic strategy. Ultimately, the externally validated trajectory-integrated nomogram may serve as a supplementary tool for bedside risk stratification, rather than a standalone predictive model.

## Supplementary material

10.2196/92838 Multimedia Appendix 1 Supplementary tables and figures. This appendix provides additional tables and figures detailing the relationship between the lactate-to-albumin ratio and clinical outcomes in critically ill patients. Contents include: temporal patterns and distribution of LAR before and after winsorization (Figure S1); restricted cubic spline analysis of the association between LAR at ICU admission (0h, 24h, 48h, 72h) and 28-day mortality (Figure S2); a web-based dynamic prognostic calculator interface (Figure S3); and supplementary tables on LAR trajectory associations, model fit statistics, baseline characteristics, and trajectory group comparisons (Tables S1–S4).

## Abbreviations

AG: anion gap
AKI: acute kidney injury
ALT: alanine aminotransferase
APACHE III: Acute Physiology and Chronic Health Evaluation III
AST: aspartate aminotransferase
C-index: concordance index
CRRT: continuous renal replacement therapy
DR: doubly robust
GBTM: group-based trajectory modeling
HF: heart failure
HIPAA: Health Insurance Portability and Accountability Act
HR: hazard ratio
ICU: intensive care unit
IDI: integrated discrimination improvement
INR: international normalized ratio
IPTW: inverse probability of treatment weighting
KDIGO: Kidney Disease Improving Global Outcomes
LAR: lactate-to-albumin ratio
MIMIC: Medical Information Mart for Intensive Care
MIMIC-IV: Medical Information Mart for Intensive Care IV
NRI: net reclassification improvement
OR: odds ratio
PT: prothrombin time
RCS: restricted cubic spline
SA-AKI: sepsis-associated acute kidney injury
SAPS II: Simplified Acute Physiology Score II
SOFA: Sequential Organ Failure Assessment
STROCSS: Strengthening the Reporting of Cohort, Cross-Sectional, and Case-Control Studies in Surgery
TBIL: total bilirubin
WBC: white blood cell
