# Supplementary material for: Dynamic Lactate-to-Albumin Ratio Trajectories and Outcomes in Sepsis-Associated Acute Kidney Injury: Evidence From MIMIC-IV and a Multicenter Chinese Cohort
Source: JMIR Med Inform. 2026 Aug 14;14:e92838. doi: 10.2196/92838 (PMC13478678; doi:10.2196/92838)
Supplement: Supplementary file 1 [file medinform-v14-e92838-s001.docx]

**Appendix Online Content**

This appendix file contains the additional tables of the experimental results for readers. The content is outlined as follows:

**Figure S1:** Temporal patterns and distribution characteristics of the LAR before and after winsorization. **(A)** Time-series trajectories of LAR (left) and LAR_winsorized (right) across time; **(B)** Boxplots illustrating the distributions of LAR (left) and LAR_winsorized (right). Abbreviations: LAR, Lactate-to-albumin ratio.

**Figure S2:** Association between LAR at ICU admission and 28-day mortality by Restricted Cubic Spline Analysis. (A) Baseline (0hour, LAR_idx1); (B) 24hour (LAR_idx2); (C) 48hour (LAR_idx3); (D) 72hour (LAR_idx4). Abbreviations: LAR, Lactate-to-albumin ratio.

**Figure S3:** Interface of the web-based dynamic prognostic calculator. The interactive application allows clinicians to input individual patient parameters (including the identified LAR trajectory class, age, SOFA score, AKI stage, and vasopressor use) to obtain an individualized, real-time estimate of the 28-day survival probability. The tool is openly accessible at https://defia123.shinyapps.io/nomogram-final/.

**Table S1:** Association of LAR trajectories with in-hospital mortality

**Table S2:** Group-based trajectory modelling fit statistics and convergence diagnostics for the external validation cohort (n=508)

**Table S3:** Baseline characteristics of patients grouped according to LAR trajectory on the external validation set

**Table S4:** Characteristics of patients assigned to the Rapid-clearance trajectory (Trajectory 2) in the MIMIC-IV and external validation cohorts.

**Figure S1.** Temporal patterns and distribution characteristics of the LAR before and after winsorization. **(A)** Time-series trajectories of LAR (left) and LAR_winsorized (right) across time; **(B)** Boxplots illustrating the distributions of LAR (left) and LAR_winsorized (right). Abbreviations: LAR, Lactate-to-albumin ratio.

**Figure S2.** Association between LAR at ICU admission and 28-day mortality by Restricted Cubic Spline Analysis. (A) Baseline (0hour, LAR_idx1); (B) 24hour (LAR_idx2); (C) 48hour (LAR_idx3); (D) 72hour (LAR_idx4). Abbreviations: LAR, Lactate-to-albumin ratio.

**Figure S3.** Interface of the web-based dynamic prognostic calculator. The interactive application allows clinicians to input individual patient parameters (including the identified LAR trajectory class, age, SOFA score, AKI stage, and vasopressor use) to obtain an individualized, real-time estimate of the 28-day survival probability. The tool is openly accessible at https://defia123.shinyapps.io/nomogram-final/.

| **Table S1. Association of LAR trajectories with In-Hospital Mortality** | | | |  |
| --- | --- | --- | --- | --- |
|  |  |  |  |  |
|  |  |  |  |  |
| Model | Trajectory Class | Odds Ratio (95% CIᵃ) | *P*-value |  |
| Multivariable Logistic Regression^b^ | Class 1 | Reference | - |  |
|  | Class 2 | 1.09 (0.73 - 1.62) | .69 |  |
|  | Class 3 | 1.81 (1.07 - 3.13) | .03 |  |
| ^a^CI: Confidence Interval. ^b^Adjusted for baseline liver disease, exogenous albumin administration, and fluid resuscitation target (≥30 mL/kg). | | | |  |
|  |  |  |  |  |
|  |  |  |  |  |
|  |  |  |  |  |

| **Table S2. Group-based trajectory modelling fit statistics and convergence diagnostics for the external validation cohort (n=508)** | | | | | | | | |  |
| --- | --- | --- | --- | --- | --- | --- | --- | --- | --- |
|  |  |  |  |  |  |  |  |  |  |
| Trajectories | Log Likelihood | AICᵃ | BICᵇ | Entropyᶜ | Min Class Size | Avg Posterior Probabilityᵈ | Populated Classes | Convergence Status |  |
| class1 | −2210.74 | 4435.48 | 4465.10 | – | – | – | 1 | Converged |  |
| class2 | −1832.61 | 3689.22 | 3739.99 | 0.930 | 48 | 0.984 | 2 | Converged |  |
| class3 | −1594.20 | 3222.40 | 3294.32 | 0.973 | 29 | 0.987 | 3 | Converged |  |
| class4 | −1528.06 | 3100.11 | 3193.18 | 0.953 | 17 | 0.976 | 4 | Converged |  |
| class5 | −1528.06 | 3110.11 | 3224.34 | 0.594 | NA | NA | 4 (1 collapsed) | Collapsed |  |
| class6 | −1528.06 | 3120.11 | 3255.49 | 0.447 | NA | NA | 4 (2 collapsed) | Failed (NA SE) |  |
| class7 | −1528.06 | 3130.11 | 3286.64 | 0.392 | NA | NA | 4 (3 collapsed) | Failed (NA SE) |  |
| ᵃAIC: Akaike Information Criterion.  ᵇBIC: Bayesian Information Criterion.  ᶜEntropy: Entropy Value.  ᵈAvgPostProb: Average Posterior Probability. Note: Fit statistics were obtained via a gridsearch with 50 random starts. The identical log-likelihood values for the four- to seven-class solutions indicate convergence to the same likelihood maximum during optimization, rather than a transcription error: specifying more than four classes resulted in one or more empty or redundant classes (class collapse), leaving only four populated classes. Thus, the higher AIC and BIC for these solutions reflect only the additional parameter penalty. The five-class solution converged with one collapsed class, whereas the six- and seven-class solutions did not fully converge (singular Hessian; SE = NA). This is a known phenomenon in lcmm-fitted latent class mixed models when data do not support further class separation. Model selection was based on information criteria, classification diagnostics (entropy and average posterior probability), minimum class size, and stability across 50 random starts; the three-class solution was retained for its adequate class separation, sufficient class sizes, parsimony, and consistency with the derivation cohort. –, not defined for a single-class model; NA, not applicable due to class collapse or non-convergence. | | | | | | | | |  |
|  |  |  |  |  |  |  |  |  |  |
|  |  |  |  |  |  |  |  |  |  |
|  |  |  |  |  |  |  |  |  |  |
|  |  |  |  |  |  |  |  |  |  |
|  |  |  |  |  |  |  |  |  |  |
|  |  |  |  |  |  |  |  |  |  |

**Table S3. Baseline characteristics of patients grouped according to LAR trajectory on the external validation set**

| Variables | Total (n = 508) | Trajectory 1 (n = 444) | Trajectory 2 (n = 35) | Trajectory 3 (n = 29) | *P* |
| --- | --- | --- | --- | --- | --- |
| **Demographics** |  |  |  |  |  |
| Age, years | 67.00 (57.00, 76.00) | 67.00 (57.00,75.25) | 68.00 (61.50,77.50) | 72.00 (46.00,78.00) | .64 |
| Gender, n(%) |  |  |  |  | .37 |
| Male | 348 (68.50) | 309 (69.59) | 21 (60.00) | 18 (62.07) |  |
| Female | 160 (31.50) | 135 (30.41) | 14 (40.00) | 11 (37.93) |  |
| **Vital Signs** |  |  |  |  |  |
| Tempᵃ, °C | 37.00 (36.50, 37.60) | 37.00 (36.50,37.60) | 37.20 (36.40,37.80) | 37.00 (36.50,37.60) | .96 |
| HRᵇ, bpm | 95.00 (83.75, 110.25) | 94.00 (82.00,109.00) | 106.00 (94.00,128.00) | 104.00 (94.00,124.00) | <.001 |
| RRᶜ, bpm | 20.00 (16.00, 24.00) | 19.50 (16.00,24.00) | 23.00 (19.00,27.00) | 21.00 (17.00,30.00) | .01 |
| SpO₂ᵈ, % | 97.30 (83.07, 115.00) | 97.45 (82.50,115.00) | 98.00 (88.45,115.00) | 95.00 (81.60,107.00) | .74 |
| **Laboratory indicators** |  |  |  |  |  |
| WBCᵉ, ×10⁹/L | 12.67 (7.59, 17.59) | 12.64 (7.74,17.47) | 12.68 (6.99,19.34) | 12.99 (5.95,16.84) | .91 |
| PLTᶠ, ×10⁹/L | 123.50 (76.00, 200.00) | 130.00 (82.75,203.00) | 82.00 (49.50,162.00) | 82.00 (38.00,123.00) | <.001 |
| INRᵍ, ratio | 1.23 (1.09, 1.38) | 1.21 (1.08,1.35) | 1.39 (1.25,1.75) | 1.32 (1.21,1.70) | <.001 |
| TBILʰ, umol/L | 21.00 (12.30, 42.78) | 20.20 (11.97,40.47) | 28.00 (13.30,56.75) | 32.30 (15.40,55.10) | .09 |
| Scrⁱ, umol/L | 141.00 (101.00, 229.80) | 140.95 (99.77,227.73) | 179.30 (112.75,242.35) | 138.00 (99.00,200.00) | .35 |
| PCTʲ, ng/ml | 4.70 (1.20, 21.00) | 3.70 (1.20,18.34) | 12.39 (3.10,30.00) | 19.80 (3.20,84.00) | .003 |
| 24-h UVᵏ, ml | 1300.00 (623.75, 2631.25) | 1397.50 (750.00,2772.50) | 1100.00 (385.00,1957.50) | 810.00 (260.00,1320.00) | .02 |
| **ICU Scoring Systems** |  |  |  |  |  |
| APACHE IIˡ, score | 19.00 (14.00, 25.00) | 18.00 (14.00,25.00) | 19.00 (14.00,23.50) | 22.00 (17.00,28.00) | .11 |
| SOFAᵐ, score | 9.00 (7.00, 12.00) | 9.00 (7.00,12.00) | 9.00 (7.50,13.00) | 13.00 (9.00,14.00) | .007 |
| **Comorbidities** |  |  |  |  |  |
| AKIⁿ, n(%) |  |  |  |  | .002 |
| 1 | 245 (48.23) | 225 (50.68) | 8 (22.86) | 12 (41.38) |  |
| 2 | 111 (21.85) | 94 (21.17) | 14 (40.00) | 3 (10.34) |  |
| 3 | 152 (29.92) | 125 (28.15) | 13 (37.14) | 14 (48.28) |  |
| CKDᵒ, n(%) | 103 (20.28) | 89 (20.05) | 6 (17.14) | 8 (27.59) | .55 |
| **Therapeutic Interventions** |  |  |  |  |  |
| Vasopressor use, n(%) | 411 (80.91) | 358 (80.63) | 30 (85.71) | 23 (79.31) | .74 |
| Ventilation, n(%) | 436 (85.83) | 378 (85.14) | 33 (94.29) | 25 (86.21) | .36 |
| CRRTᵖ, n(%) | 196 (38.58) | 160 (36.04) | 18 (51.43) | 18 (62.07) | .006 |
| ᵃTemp: Temperature.  ᵇHR: Heart Rate.  ᶜRR: Respiratory Rate.  ᵈSpO₂: Peripheral Oxygen Saturation.  ᵉWBC: White Blood Cells.  ᶠPLT: Platelets.  ᵍINR: International Normalized Ratio.  ʰTBIL: Total Bilirubin.  ⁱScr: Serum Creatinine.  ʲPCT: Procalcitonin.  ᵏ24-h UV: 24-hour urine volume.  ˡAPACHE II: Acute Physiology and Chronic Health Evaluation II.  ᵐSOFA: Sequential Organ Failure Assessment.  ⁿAKI: Acute Kidney Injury.  ᵒCKD: Chronic Kidney Disease.  ᵖCRRT: Continuous Renal Replacement Therapy. | | | | | |

| **Table S4. Characteristics of patients assigned to the Rapid-clearance trajectory (Trajectory 2) in the MIMIC-IV and external validation cohorts** | | | | |  |
| --- | --- | --- | --- | --- | --- |
|  |  |  |  |  |  |
| Variables | MIMIC-IV Cohort (n = 129) | Validation Cohort (n = 35) | *P* value | SMD |  |
| Age, years, mean (SD) | 59.02 (15.85) | 66.94 (15.34) | .009 | 0.508 |  |
| SOFA^a^ score, mean (SD) | 5.91 (3.08) | 10.03 (4.62) | < .001 | 1.047 |  |
| AKI^b^ stage, n (%) |  |  | < .001 | 0.744 |  |
| 1 | 8 (6.2) | 8 (22.9) |  |  |  |
| 2 | 30 (23.3) | 14 (40.0) |  |  |  |
| 3 | 91 (70.5) | 13 (37.1) |  |  |  |
| Vasopressor use, n (%) | 68 (52.7) | 30 (85.7) | .001 | 0.765 |  |
| Mechanical ventilation, n (%) | 114 (88.4) | 33 (94.3) | .48 | 0.211 |  |
| CRRT^c^ initiation, n (%) | 44 (34.1) | 18 (51.4) | .09 | 0.356 |  |
| 28-day mortality, n (%) | 35 (27.1) | 13 (37.1) | .35 | 0.216 |  |
| ^a^SOFA: Sequential Organ Failure Assessment. ^b^AKI: Acute Kidney Injury. ^c^CRRT: Continuous Renal Replacement Therapy. | | | | |  |
|  |  |  |  |  |  |
|  |  |  |  |  |  |
